# Clinical global assessment of nutritional status as predictor of mortality in chronic kidney disease patients

**DOI:** 10.1371/journal.pone.0186659

**Published:** 2017-12-06

**Authors:** Lu Dai, Hideyuki Mukai, Bengt Lindholm, Olof Heimbürger, Peter Barany, Peter Stenvinkel, Abdul Rashid Qureshi

**Affiliations:** 1 Division of Renal Medicine and Baxter Novum, Department of Clinical Sciences, Intervention and Technology, Karolinska Institutet, Stockholm, Sweden; 2 Renal Department, First Affiliated Teaching Hospital, Tianjin University of Traditional Chinese Medicine, Tianjin, China; Leibniz-Institut fur Pflanzengenetik und Kulturpflanzenforschung Gatersleben, GERMANY

## Abstract

**Background:**

The value of subjective global assessment (SGA) as nutritional assessor of protein-energy wasting (PEW_SGA_) in chronic kidney disease (CKD) patients depends on its mortality predictive capacity. We investigated associations of PEW_SGA_ with markers of nutritional status and all-cause mortality in CKD patients.

**Methods:**

In 1031 (732 CKD1-5 non-dialysis and 299 dialysis) patients, SGA and body (BMI), lean (LBMI) and fat (FBMI) body mass indices, % handgrip strength (% HGS), serum albumin, and high sensitivity C-reactive protein (hsCRP) were examined at baseline. The five-year all-cause mortality predictive strength of baseline PEW_SGA_ and during follow-up were investigated.

**Results:**

PEW_SGA_ was present in 2% of CKD1-2, 16% of CKD3-4, 31% of CKD5 non-dialysis and 44% of dialysis patients. Patients with PEW_SGA_ (n = 320; 31%) had higher hsCRP and lower BMI, LBMI, FBMI, %HGS and serum albumin. But, using receiver operating characteristics-derived cutoffs, these markers could not classify (by kappa statistic) or explain variations of (by multinomial logistic regression analysis) presence of PEW_SGA_. In generalized linear models, SGA independently predicted mortality after adjustments of multiple confounders (RR: 1.17; 95% CI: 1.11–1.23). Among 323 CKD5 patients who were re-assessed after median 12.6 months, 222 (69%) remained well-nourished, 37 (11%) developed PEW_SGA_ de novo, 40 (12%) improved while 24 (8%) remained with PEW_SGA_. The latter independently predicted mortality (RR: 1.29; 95% CI: 1.13–1.46).

**Conclusions:**

SGA, a valid assessor of nutritional status, is an independent predictor of all-cause mortality both in CKD non-dialysis and dialysis patients that outperforms non-composite nutritional markers as prognosticator.

## Introduction

Poor nutritional status due to protein-energy wasting (PEW) is a common complication [1–3] associated with increased mortality in patients with chronic kidney disease (CKD) [4–7]. Most nutritional markers used in clinical practice are influenced by CKD, co-morbidities [8, 9] or non-nutritional factors such as inflammation, overhydration, and protein losses [10–13]. Furthermore, their mortality predictive capacity may be skewed; for example, higher levels of BMI [14–16] and serum lipids [17] that associate with poor outcomes in the general population may predict improved survival in CKD, a phenomenon referred to as reverse epidemiology.

Subjective global nutritional assessment (SGA) is a practical, non-invasive and inexpensive composite tool that is widely used in clinical practice [18]. The concurrent and mortality predictive validity of the SGA score system has been established in conservatively treated CKD patients and incident and prevalent dialysis patients [8, 19, 20]. However, SGA of non-dialysis-dependent CKD patients [21] and the mortality predictive role of temporal changes in SGA [22, 23] have with some exceptions been less thoroughly investigated.

SGA is thought to give a valid composite measure of nutritional status in CKD patients; however, its value as a nutritional assessor depends on its mortality predictive capacity. Therefore, we evaluated SGA in patients with different stages of CKD and different dialysis modalities, explored factors classifying presence of PEW as assessed by SGA (PEW_SGA_), and analyzed the association of PEW_SGA_ with all-cause mortality.

## Methods

### Study patients

In this *post hoc* analysis we used SGA data from 1031 CKD patients including 83 CKD stage 1–2, 101 CKD stage 3–4, and 548 non-dialyzed CKD stage 5 (CKD5-ND) patients, and 299 prevalent dialysis (CKD5-D) patients, 212 hemodialysis (HD) and 87 peritoneal dialysis(PD) patients, from six cohorts, the details of which were described previously [8, 24–27]. We determined the prevalence of PEW_SGA_ and analyzed associations of PEW_SGA_ with nutritional markers at baseline and with subsequent 5 years all-cause mortality. Analyses were repeated for 323 CKD 5 non-dialysis (CKD 5-ND) patients who were re-assessed with SGA after median 12.6 months. The flow of the study subjects is described in S1 Fig.

Informed written consent was obtained from each individual. The Ethics Committee of the Karolinska Institute (EPN) at the Karolinska University Hospital Huddinge, Stockholm, Sweden, approved study protocols. The studies were conducted in adherence to the Declaration of Helsinki.

Included cohorts are described briefly, as follows:

*CKD stage 1–2*. 83 individuals from PRIMA controls cohort, a population-based sample randomly selected by Statistics Sweden (a government agency) from the Stockholm region, and recruited from February 2003 until May 2013, and who were found to have signs of mild CKD (macro- or microalbuminuria or reduced glomerular filtration rate, GFR). This cohort was created to provide an appropriate control group for the PRIMA cohort with similar age and gender distribution, see below. The median age was 61 years, 70% were males, 8% had diabetes and 8% had cardiovascular disease (CVD). Their median (10th to 90th percentile) estimated GFR (eGFR) was 85.5 (68.5–109.0) ml/min/1.73^2^.

*CKD stage 3–4*. 101 CKD stage 3–4 patients from the PRIMA cohort [28] recruited from December 1996 until November 2014. Their median age was 59 years, 72% were males, 39% had diabetes and 35% had CVD. The most common causes of CKD were glomerulonephritis (26%), diabetic nephropathy (19%), hypertension/renal vascular disease (4%) and others (51%). Their median eGFR was 27.9 (16.7–46.5) ml/min/1.73^2^.

*CKD5-ND*. 548 CKD5-ND patients (501 CKD5-ND patients initiating dialysis therapy from MIA cohort [29] and 47 patients undergoing living donor renal transplantation, LD-Rtx cohort [30]) were included in the study, and recruited from June 1994 until June 2016. Their median age was 55 years, 63% were males, 30% were diabetics, 25% had CVD and 31% were malnourished (SGA>1), and median eGFR was 6.3 (4.0–10.3) ml/min/1.732. The common causes of CKD were glomerulonephritis (26%), diabetic nephropathy (26%), hypertension/renal vascular disease (21%) and others (28%).

*CKD5-D*. 299 prevalent dialysis patients were recruited from MIMICK1 (24), (Mapping of Inflammation Markers in Chronic Kidney Disease 1), MIMICK2 [31] (Mapping of Inflammation Markers in Chronic Kidney Disease 2) and LD-Rtx [32] cohort from October 2003 to June 2016. Altogether 212 patients (71%) were treated by HD (174 from MIMICK1, 38 from LD-Rtx) and 87 patients (29%) were on PD (51 from MIMICK2, 36 from LD-Rtx). Their median age was 62 years, 60% were males, 19% had diabetes, 45% had CVD, 44% were malnourished (SGA score >1), and median eGFR was 0 (0–5.3) ml/min/1.73^2^. Causes of CKD included glomerulonephritis (18%), diabetic nephropathy (11%), hypertension/renal vascular disease (18%) and others (53%).

### Collection of clinical data

Each patient’s medical chart was reviewed to extract data pertaining to underlying etiology of CKD and co-morbidities as described previously [8, 28, 30, 32, 33].

### Assessment of nutritional status by SGA

Nutritional status was assessed using the 4-point SGA scale consisting of six components: three based on the patient’s history of weight loss, incidence of anorexia and vomiting, and three based on the physician’s grading of muscle wasting, presence of edema and loss of subcutaneous fat as described previously [1]. PEW_SGA_ was defined as SGA score >1 while a score of 1 indicated normal nutritional status. The 323 CKD 5 patients who were re-assessed with SGA after median 12.6 months were classified into four groups according to changes in nutritional status: Group 1 _WN-WN_, patients with a stable status of being well-nourished; Group 2 _MN-WN_, patients who improved their nutritional status; Group 3 _WN-MN_, patients with worsening nutritional status; Group 4 _MN-MN_, patients with PEW_SGA_ at baseline and at follow-up.

### Anthropometric evaluation

At the time of recruitment, body weight, BMI (kg/m^2^), and other anthropometric measurements were obtained. Skinfold thickness was measured with a Harpenden caliper at four sites on the non-dominant arm of the controls and in the fistula-free arm of the CKD patients. Lean body mass and fat mass were calculated by anthropometry with measurements of biceps, triceps, sub-scapular and supra-iliac skinfold thickness using the Durnin and Womersley caliper method [34], and by equations proposed by Siri [35]. Lean (LBMI) and fat (FBMI) body mass indices were calculated according to the method of Kyle et al [36] and expressed as kg/m^2^. Handgrip strength (HGS) was measured both in the dominant and non-dominant hands by using a Harpenden Handgrip Dynamometer (Yamar, Jackson, MI, USA). Each measurement was repeated three times for each arm, and the highest value for each arm was noted. Individuals in the CKD 1–2 cohort served as controls, and HGS values of CKD 3–5 patients were converted into percentage of the controls (% HGS).

### Laboratory analysis

Blood samples were collected after an overnight fast (except for HD patients). The plasma was separated within 30 min, and samples were kept frozen at -70°C if not analyzed immediately. Plasma concentrations of insulin-like growth factor-1 (IGF-1), interleukin -6 (IL-6) and tumor necrosis factor alpha (TNF-α) were measured on an Immulite TM Automatic Analyzer (Siemens Healthcare; Diagnostics Products Ltd.) according to the manufacturer’s instructions. Concentrations of serum creatinine, serum albumin (bromcresol purple), calcium, phosphate, intact parathyroid hormone (iPTH), cholesterol, triglyceride (TG), hemoglobin, hsCRP (high-sensitivity nephelometry assay) were measured by routine methods at the Department of Laboratory Medicine, Karolinska University Hospital at Huddinge.

GFR was assessed in CKD 5-ND (n = 548) and PD (n = 87) patients by the mean of renal urea and creatinine clearances from a 24-hour urine collection, in CKD stage 1–2 and CKD stage 3–4 patients GFR by iohexol clearance, while in HD patients who in general had no or minimal renal function GFR was assumed to be zero. For comparative reasons, GFR in all patients (except HD patients) were also estimated by the Chronic Kidney Disease Epidemiology Collaboration (CKD-EPI) formula [37].

### Statistical analyses

All variables are expressed as median (10^th^ and 90^th^ percentile) or percentage, or relative risk ratio (95% CI, confidence intervals), as appropriate. Statistical significance was set at the level of P<0.05. Comparisons between two groups were assessed with the non-parametric Wilcoxon test for continuous variables and Fischer exact test for nominal variables. Differences between three or more groups were analyzed by Kruskal-Wallis test. Univariate Spearman’s rank correlation was used to determine correlations between PEW_SGA_ and other variables. The agreement between PEW_SGA_ and other nutritional markers was evaluated by Kappa coefficient test. Receiver operating characteristics (ROC) derived area under the curve (AUC) values were used as cutoffs for analyses by multinomial logistic regression and generalized linear model (GENMOD procedure). A multinomial logistic regression model was used to assess the strength, expressed as pseudo-r, by which various factors could ascertain the presence of baseline PEW_SGA_. We used Kaplan-Meier and Tukey-Kramer test for multiple comparison between groups. Multivariable GENMOD regression was used to analyze all-cause mortality risk at baseline and during follow-up following adjustments for age, gender, diabetes mellitus, cardiovascular disease (CVD), % HGS, LBMI, albumin, hsCRP, calendar year and treatment modality. A multiple imputation of missing values was performed using the function PROC MI, with all variables in the covariate section used to produce the values for imputation. The original n for each variable is given throughout. The results for each imputation were generated using PROC GENMOD and PHREG and then, combined using PROC MIANALYZE. We used five imputed datasets for this study to ensure that our effect estimates were not overly inaccurate because of Monte Carlo variability. All statistical analyses were performed using statistical software SAS version 9.4 (SAS Campus Drive, Cary, NC, USA).

## Results

### Clinical and biochemical characteristics

Characteristics of the 1031 patients are shown in Table 1. According to SGA, PEW_SGA_ (SGA>1) was present in 320 patients (31%) while 711 (69%) patients were well-nourished. PEW_SGA_ patients were older, more prone to be women (PEW_SGA_ 37% vs 28% among men), more often smokers, on dialysis, diabetic and with CVD, and had higher hsCRP, IL-6, TNF and iPTH, while eGFR, %HGS, BMI, LBMI, FBMI, serum albumin, hemoglobin and IGF-1 were lower.

**Table 1:** Baseline demographic and biochemical characteristics of 1031 CKD patients according to presence of malnutrition defined as SGA score >1.

Variables	Well-nourished (n = 711)	Malnourished (n = 320)	P value
**Demography**			
Age (years)	56 (33–73)	61 (38–76)	**0.0001**
Gender, male (%)	474(67)	183(57)	**0.004**
Diabetes mellitus, n (%)	170(24)	99(31)	**0.02**
CVD, n (%)	202(28)	168(53)	**<0.0001**
Smoking, n (%) (n = 590/281)	284(48)	178(63)	**<0.0001**
Cause of kidney disease, n (%):			**<0.0001**
Glomerulonephritis	172(26)	53(17)	**0.0007**
Diabetic nephropathy	115(18)	77(24)	**0.02**
Hypertension/Renal vascular disease	105(16)	66(21)	0.07
Unknown or other etiology	243(37)	122(38)	0.69
eGFR (ml/min/1.73^2^) ^a^	6.1 (0–68.8)	5.6 (0–11.4)	**<0.0001**
Mean BP (mmHg; n = 644/268)	106 (88–124)	102 (83–128)	**0.02**
Dialysis, n (%)	168(24)	131(41)	**<0.0001**
**Anthropometric measurements**			
% HGS (n = 688/297)	93 (58–119)	67 (37–102)	**<0.0001**
BMI (kg/m^2^)	25.4 (20.9–30.9)	22.8 (18.4–29.5)	**<0.0001**
LBMI (kg/m^2^; n = 620/270)	17.6 (14.3–20.7)	16.0 (13.4–19.9)	**<0.0001**
FBMI (kg/m^2^; n = 620/270)	7.6 (4.5–11.7)	6.1 (3.4–10.5)	**<0.0001**
**Biochemical parameters**			
Creatinine (μmol/L)	664 (95–1017)	627 (403–917)	0.79
S-Albumin (g/L)	36 (29–41)	33 (25–39)	**<0.0001**
Calcium(mmol/L; n = 690/301)	2.4(2.1–2.5)	2.4(2.1–2.8)	**0.02**
Phosphate (mmol/L; n = 690/301)	1.6(0.9–2.5)	1.7(1.2–2.6)	**0.03**
Ca×PO_4_ (mmol^2^/L^2^; n = 690/301)	3.9 (2.2–6.1)	4.2 (2.7–6.4)	**0.007**
iPTH (ng/l; n = 632/281)	171 (34–541)	210 (41–607)	**0.03**
Cholesterol (mmol/L; n = 707/318)	4.7 (3.3–6.5)	4.6 (3.3–7.1)	0.42
TG (mmol/L; n = 703/319)	1.6 (0.8–3.3)	1.6 (0.8–2.9)	0.64
IGF-1 (μg/ml;n = 562/251)	171 (88–320)	150 (66–297)	**0.0008**
Hemoglobin (g/L)	115 (93–143)	110 (91–128)	**<0.0001**
hsCRP (mg/L)	2.5 (0.5–17)	7.3 (0.7–45.8)	**<0.0001**
IL-6 (pg/ml; n = 626/290)	4.2 (1.0–12.3)	7.8 (2.0–22.8)	**<0.0001**
TNF (pg/ml; n = 588/275)	11.2 (5.7–19.1)	13.6 (7.7–17.4)	**<0.0001**

Data presented as median (10^th^–90^th^ percentile), number or percentage. Abbreviations: SGA, subjective global assessment; CVD, cardiovascular disease; eGFR, estimated glomerular filtration rate; BP, blood pressure; % HGS, handgrip strength as percentage of the controls; BMI, body mass index; LBMI, lean body mass index; FBMI, fat body mass index; S-Albumin, serum albumin; Ca×PO_4_, calcium phosphate product; iPTH, intact parathyroid hormone; TG, triglyceride; IGF-1, insulin-growth like factor-1; hsCRP, high sensitivity C-reactive protein; IL-6, interleukin-6; TNF, tumor necrosis factor.

^a^In hemodialysis patients (HD) who in general had no or minimal renal function, eGFR was assumed to be zero; eGFR in all patients (except HD patients) were estimated by the Chronic Kidney Disease Epidemiology Collaboration (CKD-EPI) formula.

The prevalence of PEW_SGA_ increased with decline of renal residual function; from 2% in CKD 1–2 to 16% in CKD 3–4 and 31% in CKD 5-ND, and was 44% in prevalent dialysis patients (S1 Table). To investigate associations of PEW_SGA_ with age (S2 Table), gender (S3 Table), co-morbidities (CVD (S4 Table) diabetes (S5 Table)), renal replacement therapy (RRT) (S6 Table), anthropometry (%HGS (S7 Table), LBMI (S8 Table), FBMI (S9 Table) BMI (S10 Table)), serum albumin (S11 Table), and inflammatory status (S12 Table), we divided the patients according to these factors. The prevalence of PEW_SGA_ increased with higher age; for age **≤** 45 years, 45–65 years and >65 years, 22%, 31% and 38%, respectively (p = 0.0003) in parallel with increased co-morbidity and inflammation, and lower %HGS and serum albumin. Females had higher prevalence of PEW_SGA_ and their %HGS, LBMI, FBMI and serum albumin levels were lower. Patients with co-morbidities had higher prevalence of PEW_SGA_, higher hsCRP, and lower %HGS and serum albumin levels. Dialysis patients had worse SGA scores, and also lower levels of %HGS, serum albumin and FBMI. Patients with lower values of anthropometric measurements (%HGS, LBMI, FBMI, BMI), all tended to have higher frequency of PEW_SGA_.

### Univariate correlations, kappa analysis and multinomial logistic regression analysis of factors associated with PEW_SGA_

In both dialysis (n = 299) and non-dialysis (n = 732) patients, PEW_SGA_ was negatively associated with % HGS, BMI, LBMI, FBMI, albumin and IGF-1, and positively associated with hsCRP and IL-6 (Table 2). In addition, PEW_SGA_ correlated with age, DM, CVD, eGFR, mean BP, iPTH and TNF in non-dialysis patients, and, in dialysis patients, with female gender. The strongest correlations in non-dialysis patients was for %HGS (rho = -0.38; p<0.001) and in dialysis patients for IL-6 (rho = 0.27; p<0.001).

**Table 2:** Univariate Spearman’s Rho correlations of SGA with other parameters in 1031 CKD patients.

	Rho correlations with SGA>1	
Variables	CKD- non dialysis (n = 732)	CKD 5- dialysis (n = 299)
Age (years)	0.10 ^b^	0.08
Gender (male/female)	-0.05	-0.16 ^b^
Diabetes mellitus, %	0.12^b^	0.03
CVD, %	0.28^c^	0.09
Smoking, % (n = 582/258)	0.12 ^b^	0.18 ^b^
eGFR (ml/min/1.73^2^)^d^	-0.08 ^a^	
Mean BP (mmHg, n = 582/224)	-0.08^a^	0.03
% HGS (n = 696/289)	-0.38 ^c^	-0.22 ^b^
BMI (kg/m^2^)	-0.26^c^	-0.26^c^
LBMI (kg/m^2^, n = 691/280)	-0.23 ^c^	-0.23 ^c^
FBMI (kg/m^2^, n = 691/280)	-0.18 ^c^	-0.18 ^b^
Creatinine (μmol/L)	0.06	-0.23 ^c^
Cholesterol (mmol/L, n = 730/295)	0.01	0.01
Triglyceride (mmol/L, n = 729/296)	0.02	-0.05
IGF1 (μg/ml, n = 535/278)	-0.13 ^b^	-0.13^a^
iPTH (ng/l, n = 698/215)	0.11 ^b^	-0.10
Ca×PO_4_ (mmol^2^/L^2^; n = 690/301)	0.07	0.08
Hemoglobin (g/L,n = 729/293)	-0.23 ^c^	-0.04
S-Albumin (g/L)	-0.26 ^c^	-0.20 ^b^
hsCRP (mmol/L)	0.27^c^	0.21 ^b^
IL-6 (pg/ml,n = 631/285)	0.24 ^c^	0.27 ^c^
TNF (pg/ml, n = 578/285)	0.18 ^c^	0.11

Abbreviations: SGA, subjective global assessment; CVD, cardiovascular disease; eGFR, estimated glomerular filtration rate; BP, blood pressure; % HGS, handgrip strength as percentage of the controls; BMI, body mass index; LBMI, lean body mass index; FBMI, fat body mass index; IGF-1, insulin-growth like factor -1; i PTH, intact parathyroid hormone; Ca×PO_4_, calcium phosphate product; S-Albumin, serum albumin; hsCRP, high sensitivity C-reactive protein; IL-6, interleukin-6; TNF, tumor necrosis factor. Significant correlations are marked:

^a^ P < 0.05,

^b^ P < 0.01,

^c^ P < 0.001

^d^In hemodialysis patients (HD) who in general had no or minimal renal function, eGFR was assumed to be zero; eGFR in all patients (except HD patients) were estimated by the Chronic Kidney Disease Epidemiology Collaboration (CKD-EPI) formula.

Kappa analysis did not show good agreement of presence of PEW_SGA_ with nutritional markers indicating that these markers were inadequate markers of nutritional status as assessed by SGA (S13 Table).

**Fig 1:**
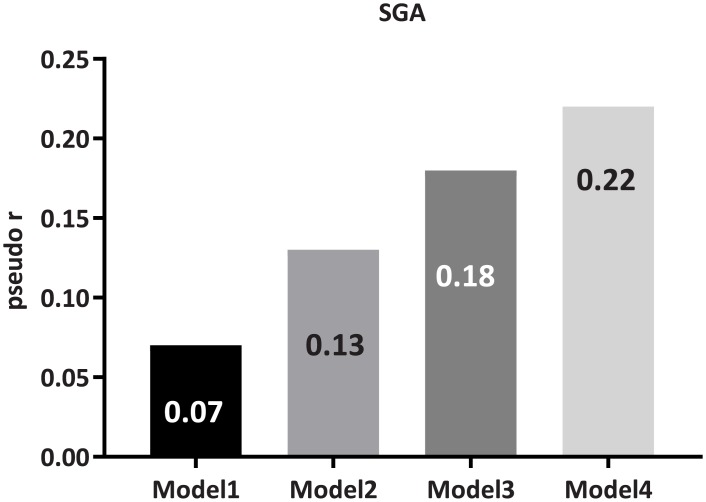
The predictive strength, expressed as pseudo-r, by which clinical and nutrition-related parameters could explain variation of presence of malnutrition by SGA. Model 1: Age, gender, diabetes mellitus and cardiovascular disease; Model 2: Model 1 + body mass index and lean body mass index; Model 3: Model 2 + high-sensitivity C-reactive protein and serum albumin; Model 4: Model 3 + handgrip strength (percentage of the controls) and treatment modality (dialysis/non-dialysis). The analysis was performed by multinomial logistic regression using cut-off values derived from receiver operating characteristics curves.

In multinomial logistic regression analysis of the strength, expressed as pseudo-r, by which various factors explained the variation of presence of PEW_SGA_, age, gender, DM and CVD together only predicted 7% of the variation of presence of PEW_SGA_ (Model 1; Fig 1). With addition of BMI and LBMI in Model 2 this increased to 13%, and to 18% after adding albumin and hsCRP in Model 3. With further addition of % HGS and RRT in Model 4, still only 22% of the variation of presence of PEW_SGA_ could be explained.

### PEW_SGA_ as independent predictor of all-cause mortality

During 5 years follow up there were 268 deaths, 8 in CKD1-2, 16 in CKD3-4, 136 in CKD 5-ND and 108 in dialysis (86 in HD and 22 in PD) patients. The relative risk of death, during the 5 years follow up, was independently associated with baseline presence of PEW_SGA_ (RR = 1.17; 95% CI, 1.11–1.23, p<0.0001) after adjustments for age, gender, DM, CVD, %HGS, LBMI, albumin, hsCRP, calendar year and RRT in 1031 patients (Table 3). In a separate analysis of the mortality predictive role of PEW_SGA_ in different strata (S14 Table) and (S15 Table), PEW_SGA_ was a predictor of all-cause mortality irrespective of whether patients were treated by dialysis (RR = 1.15; 95% CI, 1.07–1.23, p<0.0001) or not (RR = 1.19; 95% CI, 1.08–1.31, p = 0.0003).

**Table 3:** All-cause mortality risk for death occurring within 60 months based on imputed baseline data in 1031 patients, adjusted for all confounders, and expressed as relative risk ratio (95% CI).

	Relative Risk Ratio (95% CI)	P value
SGA>1, malnourished versus well nourished	1.17 (1.11–1.23)	**<0.0001**
Age, > 61 versus <61 years ^a^	1.14 (1.09–1.20)	**<0.0001**
Gender, male versus female	1.08 (1.02–1.14)	**0.01**
Diabetes mellitus, presence versus absence	1.11 (1.05–1.17)	**0.0002**
CVD (yes/no),presence versus absence	1.12 (1.06–1.18)	**<0.0001**
% HGS, >74.07 versus <74.07^a^	1.19 (1.13–1.25)	**<0.0001**
LBMI, >17 versus < 17 kg/m^2^ ^a^	1.06 (1.01–1.12)	**0.03**
Albumin, >34 versus <34 g/L^a^	1.06 (1.01–1.11)	**0.02**
hsCRP, > 4.7 versus < 4.7 mg/L^a^	1.07 (1.02–1.12)	**0.01**
Calendar year, 1994–1999 vs 2010–2016	1.16 (1.07–1.25)	**0.0003**
Calendar year, 2000–2004 vs 2010–2016	1.17 (1.10–1.24)	**<0.0001**
Calendar year, 2005–2009 vs 2010–2016	1.16 (1.08–1.25)	**<0.0001**
Dialysis vs Non dialysis	1.06 (1.01–1.12)	**0.03**

Abbreviations: 95% CI, 95% confidence interval; SGA, subjective global assessment; CVD, cardiovascular disease; % HGS, handgrip strength as percentage of the controls; LBMI, lean body mass index; hsCRP, high sensitivity C-reactive protein.

^a^ Cut-offs defined by ROC curve analysis.

### Mortality risk of PEW_SGA_ stratified by inflammation status

When patients were stratified into four groups according to presence or absence of inflammation (hsCRP≥ 4.7 mg/L; cutoff derived from ROC) and PEW_SGA_ respectively, the survival rates by Kaplan-Meier estimates differed significantly (p<0.0001; Log-Rank test), S2 Fig. Inflamed patients with PEW_SGA_ had the worst clinical outcome (p<0.0001). For both well-nourished and PEW_SGA_ patients, those with an inflamed status had higher mortality risk (both P<0.0001). On the other hand, well-nourished patients tended to have survival advantage over PEW_SGA_ patients irrespective of inflammation.

### Changes in PEW_SGA_ variation and nutritional parameters during follow up

After median 12.6 months follow up of 323 CKD 5-ND patients investigated prior to dialysis initiation, 222 (69%; Group 1 _WN-WN_) patients remained well nourished, 40 (12%; Group 2 _MN-WN_) improved, 37 (11%; Group 3 _WN-MN_) developed PEW_SGA_ de novo, and 24 (8%; Group 4 _MN-MN_) remained with PEW_SGA_ (Table 4). Serum albumin rose and hsCPR decreased in those with improving nutritional status (Group 2 _MN-WN_) while no significant changes in BMI, %HGS, LBMI or FBMI were observed during the follow up among any of the four groups of patients.

**Table 4:** Nutritional markers at baseline and after a median follow-up of 12.6 months in four groups^a^ defined by changes in SGA.

	Group _WN-WN_ (n = 222)	Group _MN-WN_ (n = 40)	Group _WN-MN_ (n = 24)	Group _MN-MN_ (n = 37)	P value
S-Albumin, baseline, g/L	36(29.0–41.0)	33(26.1–38.9)	33.5(25.0–40.5)	33(24.8–41.0)	**<0.0001**
S-Albumin, after follow-up, g/L (n = 213/38/22/34)	37(31.4–43.0) ^b^	35(30.6–41.1)^c^	36(26.0–41.1)	34(26.0–42.0)	**0.005**
hsCRP, baseline, mg/L	3.4(0.6–15.7)	11.5(1.4–50.8)	6.4(0.4–20)	6.2(0.8–31.8)	**0.0002**
hsCRP, after follow-up, mg/L (n = 210/38/20/32)	2.8(0.5–15.3)	4.5(0.8–23.7) ^c^	2.6(0.6–126.2)	5.8(0.2–47.3)	0.06
Cholesterol, baseline, mmol/L (n = 221/40/24/37)	4.9(3.5–6.9)	5.3(3.4–7.7)	5.3(3.1–6.4)	4.9(3.3–7.7)	0.54
Cholesterol, after follow-up, mmol/L (n = 214/38/22/34)	5.3(3.6–7.4)	5.5(3.8–8.5)	4.9(2.9–7.8)	5.2(3.8–7.2)	0.20
%HGS baseline (n = 215/39/22/36)	93.0(66.0–126.6)	74.1(55.6–103.7)	74.4 (50.3–108.7)	69.8(46.5–89.4)	**<0.0001**
%HGS, after follow-up, (n = 221/40/24/36)	95.3(66.8–125.9)	79.1(64.1–113.4)	74.8(51.2–115.1)	67.1(38.8–96.7)	**<0.0001**
BMI, baseline, kg/m^2^	25.7(21.3–31.4)	23.4(19.5–32.4)	24.9(18.7–31.0)	21.4(17.4–27.3)	**<0.0001**
BMI, after follow-up, kg/m^2^ (n = 222/40/24/36)	25.4(21.5–31.6)	24.7(21.0–31.9)	23.2(17.8–27.4)	21.5(16.7–27.4)	**<0.0001**
LBMI, baseline, kg/m^2^ (n = 193/31/18/29)	17.4(14.4–20.5)	16.9(13.8–21.0)	16.5(13.1–19.0)	14.9(12.0–17.4)	**<0.0001**
LBMI, after follow-up, kg/m^2^ (n = 179/29/19/29)	17.1(14.5–20.4)	16.4(14.0–18.8)	15.8(12.1–17.6)	14.2(11.4–16.6)	**<0.0001**
FBMI, baseline, kg/m^2^ (n = 193/31/18/29)	8.0(4.7–12.0)	7.5(4.2–14.3)	7.3(3.6–13.7)	6.0(3.8–9.8)	**0.005**
FBMI, after follow-up, kg/m^2^ (n = 179/29/19/29)	7.9(5.0–11.0)	7.8(5.5–12.7)	7.3(3.4–10.0)	6.5(3.3–11.5)	0.05

Data are presented as medians (range of 10^th^–90^th^ percentiles). Abbreviations: S-Albumin, serum albumin; hsCRP, high sensitivity C-reactive protein; % HGS, handgrip strength as percentage of controls; BMI, body mass index; LBMI, lean body mass index; FBMI, fat body mass index.

^a^ Groups were defined as: Group _WN-WN_, patients who remained well-nourished during follow up; Group _MN-WN_, patients who were improved with nutritional status during follow up; Group _WN-MN_, patients who developed PEW _SGA_ during the follow-up; Group _MN-MN_, patients who remained with PEW _SGA_ at baseline and at follow-up.

^b^ compared with baseline level, p<0.001;

^c^ compared with baseline level, p<0.05

### All-cause mortality associated with presence of PEW_SGA_ during follow-up

**Fig 2:**
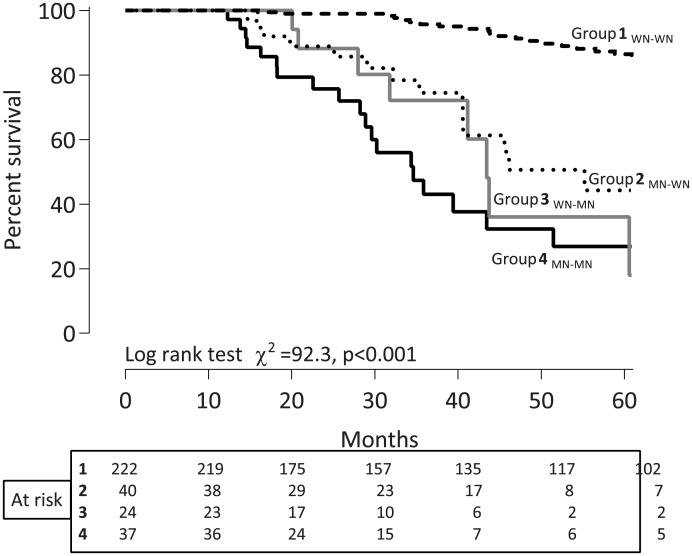
Kaplan—Meier plot for all-cause mortality of the four groups of patients according to the nutritional status change assessed by SGA. Abbreviations: Group 1 _WN-WN_, patients who remained well-nourished during follow up; Group 2 _MN-WN_, patients who were improved with nutritional status during follow up; Group 3 _WN-MN_, patients who developed PEW_SGA_ during the follow-up; Group 4 _MN-MN_, patients who remained with PEW_SGA_ at baseline and at follow-up.

When investigating the association of nutritional changes with subsequent 5 years all-cause mortality, Kaplan-Meier curves showed marked differences (P<0.0001) in 5-year survival rates between the four groups: 91% (Group 1 _WN-WN_), 65% (Group 2 _MN-WN_), 67% (Group 3 _WN-MN_), and 49% (Group 4 _MN-MN_), respectively (Fig 2). All-cause mortality of patients with persistent PEW_SGA_ (Group 1 _MN-MN_) was significantly higher compared with the other groups (P<0.0001) and also patients who developed PEW (Group 3 _WN-MN_) had better survival than those with PEW_SGA_ at both occasions (Group 4 _MN-MN_) (P = 0.002). In a separate analysis using multivariable GENMOD regression (Table 5), relative risk ratios for all-cause mortality adjusted for all investigated confounders showed that presence of PEW_SGA_ at baseline and at follow-up was an independent risk factor for 5-year death (RR = 1.29, 95% CI, 1.13–1.46, P = 0.0001).

**Table 5:** All-cause mortality risk for death occurring within 60 months based on imputed follow-up^a^ data in 323 incident dialysis patients, adjusted for all confounders, and expressed as relative risk ratio (95% CI).

	Relative Risk Ratio (95% CI)	P value
Group 2 _MN-WN_ versus Group 1 _WN-WN_	1.13(1.00–1.27)	0.05
Group 3 _WN-MN_ versus Group 1 _WN-WN_	1.15(1.00–1.33)	0.05
Group 4 _MN-MN_ versus Group 1 _WN-WN_	1.29(1.13–1.46)	**0.0001**
Age> 60 versus <60 years ^b^	1.08(0.99–1.17)	0.08
Gender, male versus female	0.98(0.90–1.07)	0.68
Diabetes mellitus, presence versus absence	1.11(1.02–1.21)	**0.02**
CVD, presence versus absence	1.09 (1.00–1.19)	0.06
% HGS, >77.78 versus <77.78^b^	1.16(1.07–1.27)	**0.0006**
LBMI >16.7 versus < 16.7 kg/m^2^ ^b^	0.98(0.89–1.07)	0.62
Albumin >33 versus <33 g/L^b^	1.10(1.02–1.19)	**0.02**
hsCRP > 6.1 versus < 6.1 mg/L^b^	1.06(0.98–1.15)	0.16
Recruitment year, 2003–2014 versus 1994–2002	0.91(0.84–0.98)	**0.02**
Hemodialysis versus peritoneal dialysis	1.02(0.95–1.10)	0.64

Abbreviations: 95% CI, 95% confidence interval; Group 1 _WN-WN_, patients who remained well-nourished during follow up; Group 2 _MN-WN_, patients who were improved with nutritional status during follow up; Group 3 _WN-MN_, patients who developed PEW_SGA_ during the follow-up; Group 4 _MN-MN_, patients who remained with PEW_SGA_ at baseline and at follow-up; CVD, cardiovascular disease; % HGS, handgrip strength as percentage of the controls; LBMI, lean body mass index; hsCRP, high sensitivity C-reactive protein

^a^ The median follow-up time of the 323 incident dialysis patients was 12.6 months.

^b^ Cut-offs defined by ROC curve analysis.

## Discussion

We found that nutritional markers (Tables 1 and 2) were associated with SGA status, but none of them nor combinations of several markers could adequately classify PEW (S13 Table) or explain variation of presence of PEW according to SGA (Fig 1), indicating that the investigated nutritional markers cannot be relied upon to ascertain presence of PEW_SGA_. On the other hand, PEW_SGA_ was an independent predictor of mortality supporting the clinical relevance and value of SGA as assessor of nutritional status in CKD patients.

SGA was found in several studies to be a reliable tool for evaluating nutritional status [19, 20, 38] and to be associated with clinical characteristics, anthropometrics and nutritional biomarkers [19, 39–42] as shown also in the present study. However, the validity of SGA as a nutritional marker has been questioned due to its subjective nature. Cooper et al [43] who compared SGA with total body nitrogen as the gold standard for protein stores in 76 dialysis patients reported that while SGA could differentiate severely malnourished patients from those with normal nutrition, SGA was not a reliable predictor of the degree of malnutrition. Jones et al [44] using a composite nutritional score derived from SGA, body mass index, percent of reference weight, triceps skinfold, mid-arm muscle circumference, and serum albumin found that SGA may not reliably identify HD patients with abnormal nutrition. On the other hand, Steiber et al [19] found that the 7-point scale SGA is a reliable and valid tool for nutritional assessment in adults on HD, and Cuppari et al [21] comparing SGA with anthropometric parameters found 7-point SGA to be a valid tool to assess PEW in nondialysis-dependent CKD patients. We concur with these more positive views on SGA but would argue that the diagnosis of malnutrition should rather be based on clinical assessment such as in the form of SGA against which the validity of proxy markers of nutritional status should be tested.

From this point of view it is interesting that PEW_SGA_ could not—based on kappa coefficient analysis—be classified adequately by several investigated single proxy markers of nutritional status including serum albumin, inflammatory biomarkers, body composition and HGS. Furthermore, the predictive strength, expressed as pseudo-r, of these markers even when used concomitantly—together with age, gender and comorbidities (CVD and DM)–to ascertain SGA status was low; collectively they could explain no more than a small fraction (pseudo-r 0.22) of the variation of presence of PEW_SGA_. Given that PEW is a complex consequence of numerous interrelated factors it is not unexpected that no single parameter can unequivocally ascertain presence of PEW. The underlying premise in the current study is thus that the diagnosis of poor nutritional status should be based on clinical assessment and that PEW_SGA_ should be regarded as a holistic clinical diagnosis. Particular features of PEW such as low muscle mass or strength are on the other hand best defined by specific markers such as LBM and HGS respectively.

In agreement with previous studies demonstrating that SGA is an independent predictor of all-cause mortality in dialysis patients [11, 39, 45], we found that PEW_SGA_ was an independent predictor of mortality not only in dialysis patients (RR = 1.15; 95% CI, 1.07–1.23, p<0.0001) but also in nondialysis-dependent patients (RR = 1.19; 95% CI, 1.08–1.31, p = 0.0003). Furthermore, in the follow-up study of 323 CKD 5 patients, PEW_SGA_ persisting during one year remained as an independent predictor for subsequent 5 year mortality risk together with only DM, %HGS, serum albumin, and recruitment period.

The strength—in spite of the subjective nature of SGA—of the association of SGA with adverse health outcomes suggests that SGA is clinically relevant. However, the link between malnutrition and increased mortality is not clear. Mutsert et al [46] found that the mortality risk of low serum albumin was partly explained by its links with inflammation, but not by malnutrition (assessed by 7-point SGA scale) in dialysis patients. In contrast, we show that PEW_SGA_ predicted all-cause mortality independent of serum albumin and inflammation (in both dialysis patients and non-dialysis patients). In addition, we also found that among patients with or without inflammation, those with normal nutrition status had lower risk of mortality than those with poor nutritional status. These results suggest that presence of PEW_SGA_ predisposes CKD patients to a worse clinical outcome, and that the mortality predictive capacity of PEW_SGA_ is not much modified by inflammation.

Furthermore, in the analyses of follow-up of changes in nutritional status, we showed that despite the variation in nutritional status by SGA, there were no significant changes of BMI, % HGS, LBMI, and FBMI among the four groups. Thus, these proxy markers of nutritional markers were not influenced by the changes in nutritional status and moreover did not—with the exception for %HGS—associate independently with the subsequent clinical outcome. In addition, although there was a significant increase of serum albumin and decrease of hsCRP concentrations in the group where nutritional status improved (Group 2 _MN-WN_), they could not fully explain the change of nutritional status since no significant variation was observed in other groups.

As expected the prevalence of PEW_SGA_ rose with the decline in GFR (from 2% in CKD 1–2, 16% in CKD 3–4, 31% in CKD 5 non-dialyzed to 44% in the dialysis patients) supplementing data from previous studies [10, 20, 41, 47]. This implies that routine monitoring of nutritional status by SGA from early stages of CKD is warranted, because not only the incidence but also severity of PEW in patients progressing into end-stage renal disease increases, and it is more difficult to treat PEW when it is severe.

The results should be interpreted considering some limitations. Firstly, because of the observational study design no conclusion can be made regarding causality and despite few missing values of nutrition-related anthropometric and biochemical parameters, we cannot rule out the impact of residual confounders. Secondly, we did not study the concurrent validity of SGA by comparing SGA with a reference method of nutritional status such as total body nitrogen and the premise of our study is that the diagnosis of PEW should be based on clinical assessment. However, the predictive validity of SGA was confirmed by the proven independent association of SGA with the risk of mortality due to poor nutritional status independent of co-morbid conditions or inflammation. Thirdly, although we evaluated nutritional status by SGA on two occasions, the stratified group sample size during follow-up was small, which may affect the association of SGA and other nutritional parameters with clinical outcome. Further studies on sequential recordings of SGA scores are warranted to verify the dynamic role of nutritional status in CKD patients.

In summary, PEW assessed by SGA was found to be an independent predictor of mortality. Our results showing that a range of non-composite nutritional markers could not adequately classify presence of clinically defined poor nutritional status, or explain the variation of SGA status, together with the finding that SGA is a robust prognosticator of clinical outcome, support the value of SGA as assessor of nutritional status in patients with CKD.

## Supporting information

S1 Fig Study cohort derivation. (PDF)

S2 Fig Kaplan—Meier plot for all-cause mortality of the four groups classified by the presence or absence of inflammation and PEW. (PDF)

S1 Table Baseline demographic and biochemical characteristics of 1031 patients according to CKD stages. (PDF)

S2 Table Comparison of CKD patients divided into different age groups. (PDF)

S3 Table Comparison of male and female CKD patients. (PDF)

S4 Table Comparison of CKD patients with and without presence of CVD. (PDF)

S5 Table Comparison of CKD patients with and without presence of diabetes mellitus. (PDF)

S6 Table Comparison of dialysis patients and non-dialysis CKD patients. (PDF)

S7 Table Comparison of CKD patients with % HGS ≥ 75 and % HGS <75. (PDF)

S8 Table Comparison of CKD patients with LBMI <19.38 (kg/m^2^) and LBMI ≥19.38 (kg/m^2^). (PDF)

S9 Table Comparison of CKD patients with FBMI <9.15 (kg/m^2^) and FBMI ≥9.15 (kg/m^2^). (PDF)

S10 Table Comparison of CKD patients with different BMI values according to division recommended by the World Health Organization. (PDF)

S11 Table Comparison of CKD patients with serum albumin <35 g/L and serum albumin ≥35 g/L. (PDF)

S12 Table Comparison of CKD patients with inflammation (hsCRP ≥10 mg/L) and without inflammation (hsCRP <10 mg/L). (PDF)

S13 Table Agreement—Expressed as kappa coefficient—of nutritional markers with presence of malnutrition (SGA score >1) at baseline. (PDF)

S14 Table All-cause mortality risk for death occurring within 60 months based on imputed baseline data in 732 CKD non-dialysis patients, adjusted for all confounders, and expressed as relative risk ratio (95% CI). (PDF)

S15 Table All-cause mortality risk for death occurring within 60 months based on imputed baseline data in 299 CKD dialysis patients, adjusted for all confounders, and expressed as relative risk ratio (95% CI). (PDF)

S1 Data Microsoft Excel database file of baseline demographic and biochemical characteristics of 1031 patients according to CKD stages. (TXT)
