# Supplementary material for: Clinical global assessment of nutritional status as predictor of mortality in chronic kidney disease patients
Source: PLoS One. 2017 Dec 6;12(12):e0186659. doi: 10.1371/journal.pone.0186659 (PMC5718431; doi:10.1371/journal.pone.0186659)
Supplement: S1 Fig — (PDF) [file pone.0186659.s001.pdf]

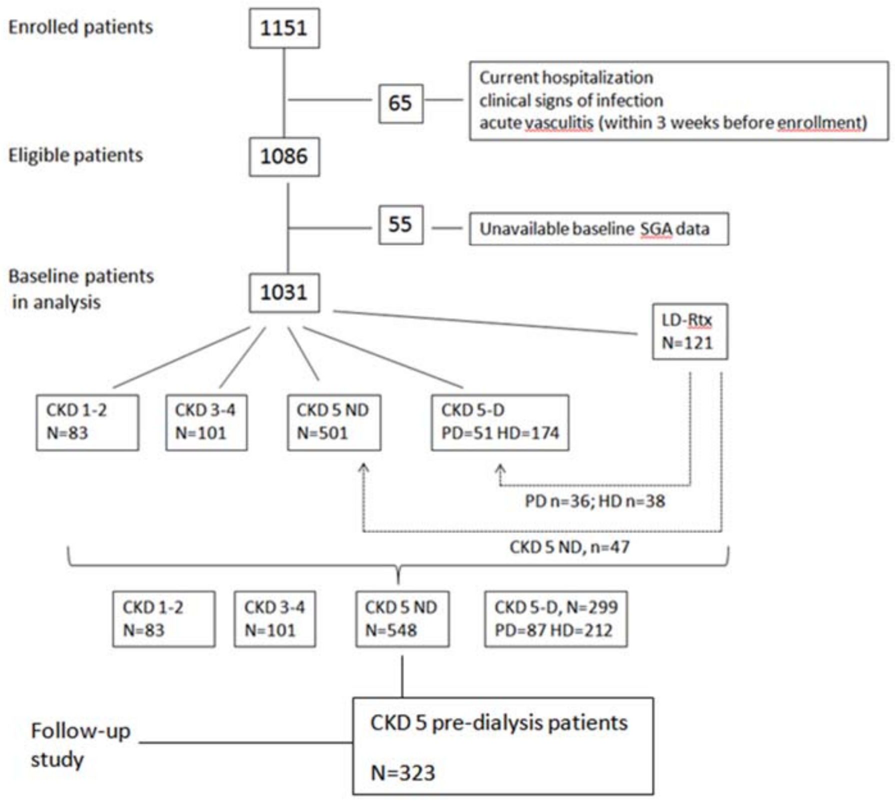

S1 Fig. Study cohort derivation

Abbreviations: CKD 5 ND, CKD 5 non-dialysis; CKD 5-D: CKD 5 dialysis dependent; LD-Rtx: Recipients of living donor renal transplant
