# Supplementary material for: Clinical global assessment of nutritional status as predictor of mortality in chronic kidney disease patients
Source: PLoS One. 2017 Dec 6;12(12):e0186659. doi: 10.1371/journal.pone.0186659 (PMC5718431; doi:10.1371/journal.pone.0186659)
Supplement: S2 Fig — (PDF) [file pone.0186659.s002.pdf]

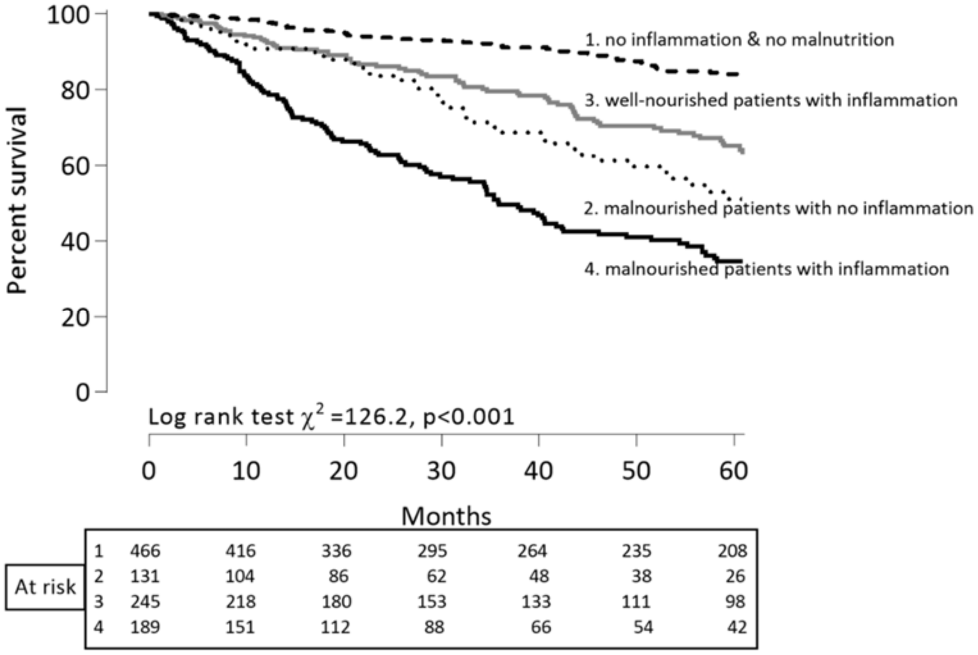

At risk							
1	466	416	336	295	264	235	208
2	131	104	86	62	48	38	26
3	245	218	180	153	133	111	98
4	189	151	112	88	66	54	42

S2 Fig. Kaplan–Meier plot for all-cause mortality of the four groups classified by the presence or absence of inflammation and PEW.
