# Supplementary material for: Clinical global assessment of nutritional status as predictor of mortality in chronic kidney disease patients
Source: PLoS One. 2017 Dec 6;12(12):e0186659. doi: 10.1371/journal.pone.0186659 (PMC5718431; doi:10.1371/journal.pone.0186659)
Supplement: S1 Table — (PDF) [file pone.0186659.s003.pdf]

S1 Table. Baseline demographic and biochemical characteristics of 1031 patients according to CKD stages.

	**CKD 1-2 (n=83)**	**CKD 3-4 (n=101)**	**CKD 5-ND (n=548)**	**CKD 5D (n=299)**	**P value**
**Demography**					
Age (years)	61 (47 – 75)	59 (36 – 74)	55 (33 - 69)	62 (34 – 80)	**<0.0001**
Gender, males (%)	58(70)	73(72)	346(63)	180(60)	0.09
Diabetes mellitus, n (%)	7(8)	39(39)	165(30)	58(19)	**<0.0001**
CVD, n (%)	7(8)	35(35)	192(35)	136(45)	**<0.0001**
Smoking, n (%) (n=78/89/446/258)	46(59)	47(53)	225(50)	144(56)	0.37
Cause of kidney disease, n (%):					**<0.0001**
Glomerulonephritis	3(4)	26(26)	141(26)	55(18)	**0.02**
Diabetic nephropathy	0	19(19)	140(26)	33(11)	**<0.0001**
Hypertension/Renal vascular disease	0	4(4)	113(21)	54(18)	**<0.0001**
Unknown or other etiology	2(2)	52(51)	154(28)	157(53)	**<0.0001**
eGFR (ml/min/1.73^2)[a]	85.5 (68.5– 109.0)	27.9 (16.7 – 46.5)	6.3 (4.0 – 10.3)	0 (0-5.3)	**<0.0001**
Mean BP(mmHg;n=83/100/505/224)	100 (88 – 120)	107 (88 – 123)	108 (90 – 126)	100 (78 – 126)	**<0.0001**
**Anthropometric measurements**					
Malnutrition, SGA>1, n (%)	2(2)	16(16)	171(31)	131(44)	**<0.0001**
%HGS (n=82/98/516/289)	100	97 (62 – 133)	84 (52 – 119)	70(42 – 108)	**<0.0001**
BMI (kg/m^2)	25.3 (20.4 – 30.0)	26.0 (20.4 – 32.0)	24.6 (19.9 – 30.7)	24.0 (19.0 – 29.8)	**0.001**
LBMI (kg/m^2; n=80/95/435/280)	17.6 (14.7-21.1)	18.1 (14.9-21.0)	16.9 (13.6-20.2)	17.0(13.8-20.5)	**0.001**
FBMI (kg/m^2; n=80/95/435/280)	7.7 (4.6-10.7)	7.6 (4.9-12.0)	7.2 (4.0 -11.3)	6.9 (3.8-11.3)	**0.03**
**Biochemical parameters**					
Creatinine (µmol/L)	0.9 (0.7- 1.1)	2.3 (1.5- 3.7)	8.0 (5.1- 11.5)	8.3 (5.6- 11.5)	**<0.0001**

S-Albumin (g/L)	39 (35 – 43)	37 (32 – 41)	34 (26 – 40)	34 (28 – 40)	**<0.0001**
Ca×PO_4 (mmol^2/L^2; n=81/98/516/296)	2.2 (1.8 – 2.7)	2.8 (2.1 – 4.1)	4.5 (3.2 – 6.4)	4.1 (2.6 – 6.3)	**<0.0001**
iPTH (ng/l ; n=75/92/531/215)	37 (24 - 62)	103 (45 - 188)	229 (52 - 595)	228 (52 - 586)	**<0.0001**
Cholesterol (mmol/L; n=83/100/547/295)	5.0 (3.9 – 6.5)	4.9 (3.8 – 6.8)	4.8 (3.2 – 7)	4.4 (3.2 – 6.2)	**<0.0001**
Triglyceride (mmol/L; n=83/99/544/296)	1.1 (0.7 – 2.6)	1.8 (0.9 – 3.6)	1.7 (0.9 – 3.4)	1.5 (0.8 – 2.8)	**<0.0001**
IGF-1 (µg/ml; n=60/78/397/278)	120 (66 – 193)	142 (68 – 230)	182 (87 – 334)	170 (82 – 318)	**<0.0001**
Hemoglobin (g/L ;n=83/100/546/293)	144 (130 – 155)	128 (105 – 148)	105 (88 – 125)	118 (99 – 134)	**<0.0001**
hs CRP (mg/L)	1.2 (0.4 – 6.4)	2.5(0.5 – 10.2)	4.0 (0.6 – 27.1)	4.1 (0.4 – 32)	**<0.0001**
IL-6 (pg/ml; n=69/52/510/285)	1.8 (0.6 – 8.5)	2.7 (1.7 – 11.9)	5.5 (1.4 – 16.7)	6.3 (1.3 – 21.4)	**<0.0001**
TNF (pg/ml; n=41/61/476/285)	3.8 (2.1 – 7.1)	7.9 (4.9 – 14.8)	11.3 (7.1 – 21.0)	14.2 (9.2 – 20.6)	**<0.0001**

Data presented as median (10th - 90th percentile), number or percentage.
Abbreviations: CVD, cardiovascular disease; eGFR, estimated glomerular filtration rate; BP, blood pressure; SGA, subjective global assessment; % HGS, handgrip strength as percentage of the controls; BMI, body mass index; LBMI, lean body mass index; FBMI, fat body mass index; S-Albumin, serum-albumin; iPTH, intact parathyroid hormone; IGF-1, insulin-growth like factor -1; hs CRP, high sensitivity C-reactive protein; IL-6, interleukin-6; TNF, tumor necrosis factor.
[a] In hemodialysis patients (HD) who in general had no or minimal renal function, eGFR was assumed to be zero; eGFR in all patients (except HD patients) were estimated by the Chronic Kidney Disease Epidemiology Collaboration (CKD-EPI) formula.
