# Supplementary material for: Clinical global assessment of nutritional status as predictor of mortality in chronic kidney disease patients
Source: PLoS One. 2017 Dec 6;12(12):e0186659. doi: 10.1371/journal.pone.0186659 (PMC5718431; doi:10.1371/journal.pone.0186659)
Supplement: S2 Table — (PDF) [file pone.0186659.s004.pdf]

S2 Table. Comparison of CKD patients divided into different age groups

	**Age≤ 45 years** (n=249)	**45<Age<65 years** (n=454)	**Age≥ 65 years** (n=328)	**P value**
**Gender, male** (%)	154 (62)	292 (64)	211 (64)	0.78
**Diabetes mellitus,** n (%)	58 (23)	122 (27)	89 (27)	0.51
**CVD**, n (%)	26 (10)	165 (36)	179 (55)	**<0.0001**
**Dialysis,** n (%)	67(27)	99(22)	133(41)	**<0.0001**
**SGA>1,** n (%)	55 (22)	142 (31)	123 (38)	**0.0003**
**% HGS** (n=232/437/316)	100(67-130)	86 (56-114)	70(44-100)	**<0.0001**
**BMI** (kg/m^2)	23.7(19.3-29.8)	24.8(20.0-31.2)	24.7(20.2-30.2)	**0.0019**
**LBMI** (kg/m^2; n=204/395/291)	17.4 (14.0-20.6)	17.1 (13.8-20.7)	17.0 (14.0-20.1)	0.10
**FBMI** (kg/m^2; n=204/395/291)	6.1 (3.0-9.6)	7.7 (4.6-11.9)	7.8 (4.7-11.1)	**<0.0001**
**S-Albumin** (g/L)	36 (28- 42)	35 (28- 41)	34 (27- 39)	**0.004**
**hsCRP** (mg/L)	1.8 (0.3-11.7)	3.7 (0.5-29.5)	4.9 (0.9-29.2)	**<0.0001**

Data presented as median (10th - 90th percentile), number and percentage.

Abbreviations: CVD, cardiovascular disease; SGA, subjective global assessment; % HGS, handgrip strength as percentage of the controls; BMI, body mass index; LBMI, lean body mass index; FBMI, fat body mass index; S-Albumin, serum-albumin; hsCRP, high sensitivity C-reactive protein
