# Supplementary material for: Clinical global assessment of nutritional status as predictor of mortality in chronic kidney disease patients
Source: PLoS One. 2017 Dec 6;12(12):e0186659. doi: 10.1371/journal.pone.0186659 (PMC5718431; doi:10.1371/journal.pone.0186659)
Supplement: S3 Table — (PDF) [file pone.0186659.s005.pdf]

S3 Table. Comparison of male and female CKD patients

	**Male** (n=657)	**Female** (n=374)	**P value**
**Age** (years)	58(36-74)	58(33-75)	0.98
**Diabetes mellitus,** n (%)	183(28)	86(23)	0.09
**CVD,** n (%)	262(40)	108(29)	**0.0004**
**Dialysis,** n (%)	180(27)	119(32)	0.13
**SGA>1,** n (%)	183 (28)	137(37)	**0.004**
**%HGS** (n=631/354)	86(50-116)	81(48-111)	**0.01**
**BMI** (kg/m^2)	24.7(20.4-27.3)	23.9(18.9-28.1)	0.05
**LBMI** (kg/m^2; n=570/320)	18.1 (15.5-21.0)	15.1 (13.1-18.4)	**<0.0001**
**FBMI** (kg/m^2; n=570/320)	6.5 (3.8-9.9)	8.6 (5.2-12.9)	**<0.0001**
**S-Albumin** (g/L)	35 (28- 41)	34 (27- 40)	**0.01**
**hsCRP** (mg/L)	3.8(0.5-26)	2.8(0.4-27)	0.08

Data presented as median (10th - 90th percentile), number and percentage.

Abbreviations: CVD, cardiovascular disease; SGA, subjective global assessment; % HGS, handgrip strength as percentage of the controls; BMI, body mass index; LBMI, lean body mass index; FBMI, fat body mass index; S-Albumin, serum-albumin; hsCRP, high sensitivity C-reactive protein
