# Supplementary material for: Clinical global assessment of nutritional status as predictor of mortality in chronic kidney disease patients
Source: PLoS One. 2017 Dec 6;12(12):e0186659. doi: 10.1371/journal.pone.0186659 (PMC5718431; doi:10.1371/journal.pone.0186659)
Supplement: S4 Table — (PDF) [file pone.0186659.s006.pdf]

S4 Table. Comparison of CKD patients with and without presence of CVD

	**Non- CVD** (n=661)	**CVD** (n=370)	**P value**
**Age** (years)	53(31-70)	64(48-78)	**<0.0001**
**Gender, male** (%)	395 (60)	262 (71)	**0.0004**
**Diabetes mellitus,** n(%)	123(19)	146 (39)	**<0.0001**
**Dialysis,** n (%)	163(25)	136 (37)	**<0.0001**
**SGA>1,** n (%)	152 (23)	168(45)	**<0.0001**
**% HGS** (n=580/318)	98(60-127)	70(38-102)	**<0.0001**
**BMI** (kg/m^2)	24.4(19.7-30.5)	24.6(20.0-30.8)	0.60
**LBMI** (kg/m^2; n=557/333)	17.2 (13.9-20.6)	17.1 (13.8-20.3)	0.54
**FBMI** (kg/m^2; n=557/333)	7.0 (4.0-11.3)	7.7 (4.5-11.5)	**0.01**
**S-Albumin** (g/L)	35(29-41)	34(26-39)	**<0.0001**
**hsCRP** (mg/L)	2.4(0.4-15.8)	6.8(0.8-39)	**<0.0001**

Data presented as median (10th - 90th percentile), number or percentage.

Abbreviations: CVD, cardiovascular disease; SGA, subjective global assessment; % HGS, handgrip strength as percentage of the controls; BMI, body mass index; LBMI, lean body mass index; FBMI, fat body mass index; S-Albumin, serum-albumin; hs CRP, high sensitivity C-reactive protein
