# Supplementary material for: Clinical global assessment of nutritional status as predictor of mortality in chronic kidney disease patients
Source: PLoS One. 2017 Dec 6;12(12):e0186659. doi: 10.1371/journal.pone.0186659 (PMC5718431; doi:10.1371/journal.pone.0186659)
Supplement: S5 Table — (PDF) [file pone.0186659.s007.pdf]

S5 Table. Comparison of CKD patients with and without presence of diabetes mellitus

	**Non- Diabetes mellitus** (n=762)	**Diabetes mellitus** (n=269)	**P value**
**Age** (years)	57(33-75)	59(37-72)	0.30
**Gender, male** (%)	474 (62)	183 (68)	0.09
**CVD,** n (%)	224 (29)	146 (54)	**<0.0001**
**Dialysis,** n (%)	241(32)	58(22)	**0.001**
**SGA>1,** n (%)	221 (29)	99(37)	**0.02**
**%HGS** (n=736/249)	89(52-117)	72(47-107)	**<0.0001**
**BMI** (kg/m^2)	24.2(19.5-30.0)	25.9(21.0-32.9)	**<0.0001**
**LBMI** (kg/m^2; n=665/225)	17.1 (13.8-20.4)	17.2 (14.1-20.6)	0.10
**FBMI** (kg/m^2; n=665/225)	6.8 (3.9-10.6)	8.4 (4.9-12.1)	**<0.0001**
**S-Albumin** (g/L)	35(29-41)	33(25-39)	**<0.0001**
**hs CRP** (mg/L)	2.9(0.5-26)	4.7(0.6-28.5)	**0.0008**

Data presented as median (10th - 90th percentile), number or percentage.

Abbreviations: CVD, cardiovascular disease; SGA, subjective global assessment; % HGS, handgrip strength as percentage of the controls; BMI, body mass index; LBMI, lean body mass index; FBMI, fat body mass index; S-Albumin, serum-albumin; hs CRP, high sensitivity C-reactive protein
