# Supplementary material for: Clinical global assessment of nutritional status as predictor of mortality in chronic kidney disease patients
Source: PLoS One. 2017 Dec 6;12(12):e0186659. doi: 10.1371/journal.pone.0186659 (PMC5718431; doi:10.1371/journal.pone.0186659)
Supplement: S6 Table — (PDF) [file pone.0186659.s008.pdf]

S6 Table. Comparison of dialysis patients and non-dialysis CKD patients

	**Dialysis** (n=299)	**Non-dialysis** (n=732)	**P value**
**Age** (years)	62(34-80)	56(35-70)	**<0.0001**
**Gender, male** (%)	180 (60)	477(65)	0.13
**Diabetes mellitus,** n (%)	58 (19)	211 (29)	**0.001**
**CVD,** n (%)	136 (46)	234 (32)	**<0.0001**
**SGA>1,** n (%)	131 (44)	189(26)	**<0.0001**
**% HGS** (n=289/697)	70 (42-108)	91 (56-119)	**<0.0001**
**BMI** (kg/m^2)	24 (19 -30)	25 (20 -31)	**0.003**
**LBMI** (kg/m^2; n=280/610)	17.0 (13.8-20.5)	17.2 (13.9-20.5)	0.14
**FBMI** (kg/m^2; n=280/610)	6.9 (3.8-11.3)	7.4 (4.4-11.5)	**0.02**
**S-Albumin** (g/L)	34 (28-40)	35 (27-41)	**0.008**
**hsCRP** (mg/L)	4.1 (0.4-32)	3.1 (0.5-24)	0.09

Data presented as median (10th - 90th percentile), number or percentage.

Abbreviations: CVD, cardiovascular disease; SGA, subjective global assessment; % HGS, handgrip strength as percentage of the controls; BMI, body mass index; LBMI, lean body mass index; FBMI, fat body mass index; S-Albumin, serum-albumin; hs CRP, high sensitivity C-reactive protein
