# Supplementary material for: Clinical global assessment of nutritional status as predictor of mortality in chronic kidney disease patients
Source: PLoS One. 2017 Dec 6;12(12):e0186659. doi: 10.1371/journal.pone.0186659 (PMC5718431; doi:10.1371/journal.pone.0186659)
Supplement: S7 Table — (PDF) [file pone.0186659.s009.pdf]

S7 Table. Comparison of CKD patients with % HGS ≥ 75 and % HGS <75

	**% HGS ≥ 75** (n=596)	**% HGS <75** (n=389)	**P value**
**Age** (years)	53(32-70)	64(44-79)	**<0.0001**
**Gender, male** (%)	398 (67)	233 (60)	**0.03**
**Diabetes mellitus,** n (%)	111 (19)	138 (35)	**<0.0001**
**CVD,** n (%)	135 (23)	216 (56)	**<0.0001**
**Dialysis,** n (%)	119(20)	170(44)	**<0.0001**
**SGA>1,** n (%)	105 (18)	192(49)	**<0.0001**
**BMI** (kg/m^2)	24.9(20.5-30.6)	23.8(19.0-30.3)	**0.0002**
**LBMI** (kg/m^2; n=528/346)	17.5 (14.5-20.9)	16.4 (13.6-19.8)	**<0.0001**
**FBMI** (kg/m^2; n=528/346)	7.1 (4.1-11.5)	7.4 (4.1-11.2)	0.61
**S-Albumin** (g/L)	36(29-41)	33(26-39)	**<0.0001**
**hsCRP** (mg/L)	2.3(0.4-16.2)	6.3(0.8-35)	**<0.0001**

Data presented as median (10th - 90th percentile), number or percentage.

Abbreviations: % HGS, handgrip strength as percentage of the controls; CVD, cardiovascular disease; SGA, subjective global assessment; BMI, body mass index; LBMI, lean body mass index; FBMI, fat body mass index; S-Albumin, serum-albumin; hsCRP, high sensitivity C-reactive protein
