# Supplementary material for: Clinical global assessment of nutritional status as predictor of mortality in chronic kidney disease patients
Source: PLoS One. 2017 Dec 6;12(12):e0186659. doi: 10.1371/journal.pone.0186659 (PMC5718431; doi:10.1371/journal.pone.0186659)
Supplement: S8 Table — (PDF) [file pone.0186659.s010.pdf]

S8 Table. Comparison of CKD patients with LBMI <19.38 (kg/m^2) and LBMI ≥19.38 (kg/m^2)

	**LBMI <19.38** (kg/m^2) (n=721)	**LBMI ≥19.38** (kg/m^2) (n=169)	**P value**
**Age** (years)	57(34-74)	59(36-75)	0.09
**Gender, male** (%)	413 (57)	157 (93)	**<0.0001**
**Diabetes mellitus,** n (%)	177 (25)	48 (28)	**0.30**
**CVD,** n (%)	270 (38)	63 (37)	0.97
**Dialysis,** n(%)	231(32)	49(29)	0.44
**SGA>1,** n (%)	234 (32)	36(21)	**0.004**
**% HGS** (n=707/167)	81 (48-112)	99 (58-128)	**<0.0001**
**BMI** (kg/m^2)	24.6(19.9-30.4)	24.4(19.5-31.2)	1.00
**FBMI** (kg/m^2)	7.9 (4.4-13.6)	7.1 (4.0-11.0)	**0.001**
**S-Albumin** (g/L)	35 (29-41)	35 (27-41)	0.14
**hsCRP** (mg/L)	3.5 (0.6-21)	3.5 (0.5-27)	0.96

Data presented as median (10th - 90th percentile), number or percentage.

Abbreviations: LBMI, lean body mass index; CVD, cardiovascular disease; SGA, subjective global assessment; % HGS, handgrip strength as percentage of the controls; BMI, body mass index; FBMI, fat body mass index; S-Albumin, serum-albumin; hsCRP, high sensitivity C-reactive protein
