# Supplementary material for: Clinical global assessment of nutritional status as predictor of mortality in chronic kidney disease patients
Source: PLoS One. 2017 Dec 6;12(12):e0186659. doi: 10.1371/journal.pone.0186659 (PMC5718431; doi:10.1371/journal.pone.0186659)
Supplement: S9 Table — (PDF) [file pone.0186659.s011.pdf]

S9 Table. **Comparison of CKD patients with FBMI <9.15 (kg/m^2) and FBMI ≥9.15 (kg/m^2)**

	**FBMI <9.15**(kg/m^2) (n=651)	**FBMI ≥9.15**(kg/m^2) (n=239)	**P value**
**Age** (years)	58(33-75)	60(43-73)	**0.0007**
**Gender, male** (%)	473 (73)	97 (41)	**<0.0001**
**Diabetes mellitus,** n (%)	136 (21)	89 (37)	**<0.0001**
**CVD,** n (%)	239 (37)	94 (39)	0.48
**Dialysis,** n (%)	209(32)	71(30)	0.49
**SGA>1,** n (%)	213 (33)	57(24)	**0.01**
**%HGS** (n=640/234)	84 (48-116)	84 (54-111)	0.72
**BMI** (kg/m^2)	23.1(19.2-27.2)	28.4(24.4-34.7)	**<0.0001**
**LBMI** (kg/m^2)	17.1 (13.7-20.2)	17.4 (14.5-21.2)	**0.0006**
**S-Albumin** (g/L)	35 (28-40)	35 (28-41)	0.34
**hsCRP** (mg/L)	4.4 (0.9-29)	3.1 (0.5-25)	**0.0008**

Data presented as median (10th - 90th percentile), number or percentage.

Abbreviations: FBMI, fat body mass index; CVD, cardiovascular disease; SGA, subjective global assessment; % HGS, handgrip strength as percentage of the controls; BMI, body mass index; LBMI, lean body mass index; S-Albumin, serum-albumin; hsCRP, high sensitivity C-reactive protein
