# Supplementary material for: Clinical global assessment of nutritional status as predictor of mortality in chronic kidney disease patients
Source: PLoS One. 2017 Dec 6;12(12):e0186659. doi: 10.1371/journal.pone.0186659 (PMC5718431; doi:10.1371/journal.pone.0186659)
Supplement: S10 Table — (PDF) [file pone.0186659.s012.pdf]

S10 Table. Comparison of CKD patients with different BMI values according to division recommended by the World Health Organization

	**Underweight** (BMI< 18.5,n=48)	**Normal** (18.5 ≤BMI≤ 24.99, n=509)	**Overweight** (25 ≤BMI< 30, n=346)	**Obese** (BMI≥ 30, n=128)	**P value**
**Age** (years)	54.5(29-80)	56(32-75)	59(38-74)	58(39-73)	0.09
**Gender, male** (%)	17 (35)	324 (64)	244 (71)	72 (56)	**<0.0001**
**Diabetes mellitus,** n(%)	7 (15)	109 (21)	98 (28)	55 (43)	**<0.0001**
**CVD,** n (%)	19 (40)	178 (35)	124 (36)	49 (38)	0.94
**Dialysis,** n (%)	22 (46)	157 (31)	92 (27)	28 (22)	**0.01**
**SGA>1,** n (%)	34 (71)	195(38)	65 (19)	26 (20)	**<0.0001**
**%HGS** (n=44/490/333/118)	64 (32-102)	81 (48-114)	89 (56-118)	86 (51-111)	**<0.0001**
**LBMI** (kg/m^2; n=42/454/301/93)	13.0 (11.6-15.2)	15.9 (13.7-18.8)	18.4 (16.1-20.8)	20.4 (17.4-24.7)	**<0.0001**
**FBMI** (kg/m^2; n=42/454/301/93)	4.3 (2.3-5.9)	6.0 (3.7-8.7)	8.7 (6.2-11.6)	14.2(9.1-16.2)	**<0.0001**
**S-Albumin** (g/L)	33(24-40)	35(27-41)	35(29-40)	34(28-40)	0.09
**hsCRP** (mg/L)	4.3(0.5-49.1)	3 (0.4-27)	3.3(0.6-24)	4.2(1.1-27.5)	**0.03**

Data presented as median (10th - 90th percentile), number or percentage.
Abbreviations: BMI, body mass index; CVD, cardiovascular disease; SGA, subjective global assessment; % HGS, handgrip strength as percentage of the controls; LBMI, lean body mass index; FBMI, fat body mass index; S-Albumin, serum-albumin; hsCRP, high sensitivity C-reactive protein
