# Supplementary material for: Clinical global assessment of nutritional status as predictor of mortality in chronic kidney disease patients
Source: PLoS One. 2017 Dec 6;12(12):e0186659. doi: 10.1371/journal.pone.0186659 (PMC5718431; doi:10.1371/journal.pone.0186659)
Supplement: S11 Table — (PDF) [file pone.0186659.s013.pdf]

S11 Table. Comparison of CKD patients with serum albumin <35 g/L and serum albumin ≥35 g/L

	**S-Albumin <35 g/L** (n=484)	**S-Albumin ≥35 g/L** (n=547)	**P value**
**Age** (years)	59(36-75)	56(32-74)	**0.01**
**Gender, male** (%)	292 (60)	365 (67)	**0.03**
**Diabetes mellitus,** n(%)	164 (34)	105 (19)	**<0.0001**
**CVD,** n (%)	204 (42)	166 (30)	**<0.0001**
**Dialysis,** n (%)	163(34)	136(25)	**0.001**
**SGA>1,** n (%)	198 (41)	122(22)	**<0.0001**
**% HGS** (n=453/533)	74 (44-111)	93 (56-116)	**<0.0001**
**BMI** (kg/m^2)	24.5(19.4-30.9)	24.5(20.2-30.4)	0.91
**LBMI** (kg/m^2; n=412/478)	17.0 (13.7-20.3)	17.3 (14.0-20.7)	0.55
**FBMI** (kg/m^2; n=412/478)	7.2 (4.0-11.7)	7.3 (4.2-11.0)	0.94
**hsCRP** (mg/L)	6.4 (0.6-42.5)	2.4 (0.4–13.0)	**<0.0001**

Data presented as median (10th - 90th percentile), number or percentage.
Abbreviations: S-Albumin, serum-albumin; CVD, cardiovascular disease; SGA, subjective global assessment; % HGS, handgrip strength as percentage of the controls; BMI, body mass index; LBMI, lean body mass index; FBMI, fat body mass index; hsCRP, high sensitivity C-reactive protein.
