# Supplementary material for: Clinical global assessment of nutritional status as predictor of mortality in chronic kidney disease patients
Source: PLoS One. 2017 Dec 6;12(12):e0186659. doi: 10.1371/journal.pone.0186659 (PMC5718431; doi:10.1371/journal.pone.0186659)
Supplement: S12 Table — (PDF) [file pone.0186659.s014.pdf]

S12 Table. **Comparison of CKD patients with inflammation (hsCRP ≥10 mg/L) and without inflammation (hsCRP <10 mg/L).**

	**hsCRP<10 mg/L** (n=773)	**hsCRP≥10 mg/L** (n=258)	**P value**
**Age** (years)	56(32-74)	61(43-75)	**<0.0001**
**Gender, male** (%)	486 (63)	171 (66)	0.32
**Diabetes mellitus,** n (%)	186 (24)	83 (32)	**0.01**
**CVD,** n (%)	224 (29)	146 (57)	**<0.0001**
**Dialysis,** n (%)	85(33)	214(28)	0.11
**SGA>1,** n (%)	189 (24)	131(51)	**<0.0001**
**%HGS** (n=744/241)	89(56-119)	70(42-103)	**<0.0001**
**BMI** (kg/m^2)	24.6(19.9-30.4)	24.4(19.5-31.2)	1.00
**LBMI** (kg/m^2; n=668/222)	17.3 (13.8-20.6)	16.8 (14.1-20.2)	0.08
**FBMI** (kg/m^2; n=668/222)	7.1 (4.1-11.0)	7.6 (4.1-12.4)	0.10
**S-Albumin** (g/L)	36(29-41)	32(25-37)	**<0.0001**

Data presented as median (10th - 90th percentile), number or percentage.
Abbreviations: hsCRP, high sensitivity C-reactive protein; CVD, cardiovascular disease; SGA, subjective global assessment; % HGS, handgrip strength as percentage of the controls; BMI, body mass index; LBMI, lean body mass index; FBMI, fat body mass index; S-Albumin, serum-albumin
