# Supplementary material for: Clinical global assessment of nutritional status as predictor of mortality in chronic kidney disease patients
Source: PLoS One. 2017 Dec 6;12(12):e0186659. doi: 10.1371/journal.pone.0186659 (PMC5718431; doi:10.1371/journal.pone.0186659)
Supplement: S13 Table — (PDF) [file pone.0186659.s015.pdf]

S13 Table. Agreement - expressed as kappa coefficient – of nutritional markers with presence of malnutrition (SGA score >1) at baseline.

parameters	Kappa coefficient	95% CI
% HGS < 74%[a]	0.31	0.25-0.37
BMI < 24.2 kg/m^2 [a]	0.23	0.17-0.28
LBMI < 17 kg/m^2 [a]	0.19	0.13-0.25
Albumin < 34 g/L [a]	0.20	0.14-0.26
hsCRP > 4.7 mg/L [a]	0.22	0.17-0.28

Abbreviations: 95% CI, 95% confidence interval; % HGS, handgrip strength as percentage of the controls; BMI, body mass index; LBMI, lean body mass index; hsCRP, high sensitivity C-reactive protein.

[a] Cut-offs defined by ROC curve analysis.

Interpretation of agreement according to kappa coefficient values: 0–0.20 indicate slight, 0.21–0.40 fair, 0.41–0.60 moderate, 0.61–0.80 substantial, and 0.81–1 almost perfect agreement.
