# Supplementary material for: Clinical global assessment of nutritional status as predictor of mortality in chronic kidney disease patients
Source: PLoS One. 2017 Dec 6;12(12):e0186659. doi: 10.1371/journal.pone.0186659 (PMC5718431; doi:10.1371/journal.pone.0186659)
Supplement: S14 Table — (PDF) [file pone.0186659.s016.pdf]

S14 Table. All-cause mortality risk for death occurring within 60 months based on imputed baseline data in 732 CKD non-dialysis patients, adjusted for all confounders, and expressed as relative risk ratio (95% CI).

	Relative Risk Ratio (95% CI)	P value
SGA>1, malnourished versus well nourished	1.15 (1.07 – 1.23)	**<0.0001**
Age, > 55 versus <55 years [a]	1.09 (1.03 – 1.15)	**0.003**
Gender, male versus female	1.03 (0.97 – 1.10)	0.32
Diabetes mellitus, presence versus absence	1.09 (1.03 – 1.16)	**0.005**
CVD (yes/no),presence versus absence	1.15 (1.08 – 1.22)	**<0.0001**
% HGS, >81.48 versus <81.48[a]	1.21 (1.14 – 1.29)	**<0.0001**
LBMI ,>19 versus < 19 kg/m^2 [a]	1.06 (0.99 – 1.12)	0.10
Albumin, >30 versus <30 g/L[a]	1.07 (1.01 – 1.13)	**0.02**
hsCRP, > 4.8 versus < 4.8 mg/L[a]	1.08 (1.02 – 1.14)	**0.007**
Calendar year, 1994- 1999 vs 2010 - 2016	1.14 (1.05 – 1.24)	**0.002**
Calendar year, 2000- 2004 vs 2010 - 2016	1.16 (1.08 – 1.25)	**<0.0001**
Calendar year, 2005- 2009 vs 2010 - 2016	1.13 (1.05 – 1.23)	**0.002**

Abbreviations: 95% CI, 95% confidence interval; CKD, chronic kidney disease; SGA, subjective global assessment; CVD, cardiovascular disease; % HGS, handgrip strength as percentage of the controls; LBMI, lean body mass index; hsCRP, high sensitivity C-reactive protein.

[a] Cut-offs defined by ROC curve analysis.
