# Supplementary material for: Clinical global assessment of nutritional status as predictor of mortality in chronic kidney disease patients
Source: PLoS One. 2017 Dec 6;12(12):e0186659. doi: 10.1371/journal.pone.0186659 (PMC5718431; doi:10.1371/journal.pone.0186659)
Supplement: S15 Table — (PDF) [file pone.0186659.s017.pdf]

S15 Table. All-cause mortality risk for death occurring within 60 months based on imputed baseline data in 299 CKD dialysis patients, adjusted for all confounders, and expressed as relative risk ratio (95% CI).

	Relative Risk Ratio (95% CI)	P value
SGA>1, malnourished versus well nourished	1.19 (1.08 – 1.31)	**0.0003**
Age, > 67 versus <67 years [a]	1.26 (1.14 – 1.40)	**<0.0001**
Gender, male versus female	1.19 (1.08 – 1.32)	**0.0007**
Diabetes mellitus, presence versus absence	1.12 (1.00 – 1.25)	0.05
CVD (yes/no),presence versus absence	1.06 (0.96 – 1.18)	0.24
% HGS, >72.09 versus <72.09[a]	1.20 (1.07 – 1.33)	**0.001**
LBMI ,>16 versus < 16 kg/m^2 [a]	1.08 (0.97 – 1.21)	0.15
Albumin, >33 versus <33 g/L[a]	1.02 (0.93 – 1.12)	0.64
hsCRP, > 2.6 versus < 2.6 mg/L[a]	1.04 (0.94 – 1.15)	0.43
Calendar year, 2000- 2004 vs 2010 - 2016	1.18 (1.04 – 1.33)	**0.01**
Calendar year, 2005- 2009 vs 2010 - 2016	1.23 (1.05 – 1.46)	**0.01**

Abbreviations: 95% CI, 95% confidence interval; CKD, chronic kidney disease; SGA, subjective global assessment; CVD, cardiovascular disease; % HGS, handgrip strength as percentage of the controls; LBMI, lean body mass index; hsCRP, high sensitivity C-reactive protein.

[a] Cut-offs defined by ROC curve analysis.
